# The meaning of significant mean group differences for biomarker discovery

**DOI:** 10.1371/journal.pcbi.1009477

**Published:** 2021-11-18

**Authors:** Eva Loth, Jumana Ahmad, Chris Chatham, Beatriz López, Ben Carter, Daisy Crawley, Bethany Oakley, Hannah Hayward, Jennifer Cooke, Antonia San José Cáceres, Danilo Bzdok, Emily Jones, Tony Charman, Christian Beckmann, Thomas Bourgeron, Roberto Toro, Jan Buitelaar, Declan Murphy, Guillaume Dumas

**Affiliations:** 1 Department of Forensic and Neurodevelopmental Sciences, Institute of Psychiatry, Psychology and Neuroscience, King’s College London, London, United Kingdom; 2 Sackler Institute for Translational Neuroscience, Institute of Psychiatry, Psychology and Neuroscience, King’s College London, London, United Kingdom; 3 Department of Psychology, Social Work and Counselling, Faculty of Education and Health, University of Greenwich, London, United Kingdom; 4 Neuroscience & Rare Diseases, Pharma Research & Early Development, Roche Innovation Center New York, New York, United States of America; 5 Department of Psychology, Portsmouth University, Portsmouth, United Kingdom; 6 Department of Biostatistics, Institute of Psychiatry, Psychology and Neuroscience, King’s College London, London, United Kingdom; 7 Instituto de Investigación Sanitaria Gregorio Marañón, Departamento de Psiquiatría del Niño y del Adolescente, Hospital General Universitario Gregorio Marañón and Centro Investigación Biomédica en Red Salud Mental (CIBERSAM), Madrid, Spain; 8 Department of Biomedical Engineering, McConnell Brain-Imaging Centre (BIC), Montreal Neurological Institute (MNI), Faculty of Medicine, McGill University, Montreal, Canada; 9 Canadian Institute for Advanced Research (CIFAR), Canada; 10 Mila–Quebec Artificial Intelligence Institute, Montreal, Canada; 11 Centre for Brain and Cognitive Development, Birkbeck, University of London, London, United Kingdom; 12 Department of Psychology, Institute of Psychiatry, Psychology and Neuroscience, King’s College London, London, United Kingdom; 13 Department of Cognitive Neuroscience, Donders Institute for Brain, Cognition and Behaviour, Radboud University Medical Centre, Nijmegen, the Netherlands; 14 Human Genetics and Cognitive Functions, Institut Pasteur, UMR3571 CNRS, Université de Paris, Paris, France; 15 Precision Psychiatry and Social Physiology laboratory, CHU Sainte-Justine Research Center, Department of Psychiatry, University of Montreal, Quebec, Canada; McGill University, CANADA

## Abstract

Over the past decade, biomarker discovery has become a key goal in psychiatry to aid in the more reliable diagnosis and prognosis of heterogeneous psychiatric conditions and the development of tailored therapies. Nevertheless, the prevailing statistical approach is still the mean group comparison between “cases” and “controls,” which tends to ignore within-group variability. In this educational article, we used empirical data simulations to investigate how effect size, sample size, and the shape of distributions impact the interpretation of mean group differences for biomarker discovery. We then applied these statistical criteria to evaluate biomarker discovery in one area of psychiatric research—autism research. Across the most influential areas of autism research, effect size estimates ranged from small (*d* = 0.21, anatomical structure) to medium (*d* = 0.36 electrophysiology, *d* = 0.5, eye-tracking) to large (*d* = 1.1 theory of mind). We show that in normal distributions, this translates to approximately 45% to 63% of cases performing within 1 standard deviation (SD) of the typical range, i.e., they do not have a deficit/atypicality in a statistical sense. For a measure to have diagnostic utility as defined by 80% sensitivity and 80% specificity, Cohen’s *d* of 1.66 is required, with still 40% of cases falling within 1 SD. However, in both normal and nonnormal distributions, 1 (skewness) or 2 (platykurtic, bimodal) biologically plausible subgroups may exist despite small or even nonsignificant mean group differences. This conclusion drastically contrasts the way mean group differences are frequently reported. Over 95% of studies omitted the “on average” when summarising their findings in their abstracts (“autistic people have deficits in X”), which can be misleading as it implies that the group-level difference applies to all individuals in that group. We outline practical approaches and steps for researchers to explore mean group comparisons for the discovery of stratification biomarkers.

## Author summary

Currently, a striking paradox is often found in neuropsychiatric research. On the one hand, most clinicians and researchers accept that many neuropsychiatric conditions involve tremendous individual variability. On the other hand, the prevailing statistical approach is still the mean group comparison between “cases” and “controls.” Statistically significant mean group differences tell us that a given characteristic in brain, behaviour, or genes is on average different between the 2 groups. Yet, they do not delineate variability within groups. Moreover, using autism research as an example, we show that in up to 95% of abstracts, when reporting or interpreting findings, researchers omit the “on average.” This can be misleading because it evokes the impression as though the group-level difference would generalise to all individuals with that condition. Here, we used simulations to show that the latter statement is only true at very large effect sizes. We demonstrate that across different areas of autism research, mean group differences with small to large effects indicate that approximately 45% to 68% [cases] do not have an atypicality on cognitive tests or brain structure. However, we also show that across normal and nonnormal distributions, subgroups may exist despite small or nonsignificant overall effects. We propose practical approaches and steps for researchers to use mean group comparisons as the starting point for the discovery of clinically relevant subgroups.

## Introduction

Currently, there is a striking paradox in neuropsychiatric research. On the one hand, the clinical and etiological heterogeneity of most neurodevelopmental and psychiatric conditions (as well as substantial overlap between conditions) is widely accepted among researchers and clinicians [1]. This means that individuals with a particular “umbrella” clinical diagnosis do not necessarily share the same neurocognitive and neurobiological characteristics [2,3]. These findings have prompted increasing interest in biomarker discovery to enable participant stratification and precision medicine approaches [4]. On the other hand, the prevailing statistical approach in neuropsychiatry remains the mean group comparison between a clinical case group A and a “neurotypical control” group B. This analysis approach is rooted in the traditional categorical framework to psychiatry that assumes that a given clinical condition involves one or more defining neurocognitive or neurobiological characteristic(s) that is (are) *universal* and *specific* to that condition [2,5]. Moreover, statistically significant mean group differences only indicate what is different between group A (e.g., Autism) and group B (e.g., a “neurotypical” comparison group) *on average*. However, those differences do not delineate variability *within* groups. Mean group differences may reflect a systematic shift in the distribution of the clinical group and thus provide useful information on altered processes in that population. However, whether or not the characteristic is indeed universal to the clinical group and accurately distinguishes cases from controls—which would be required for the measure to have diagnostic utility—or may only apply to a subset of individuals depends on the strength of the separation between the distributions [6].

In this educational article, we consider how effect size, sample size, and the shape of the distributions impact the interpretation of mean group differences for the discovery of clinically useful biomarkers in biomedical research.

## Biomarkers in neuropsychiatry

Broadly, a biomarker has been defined as “a characteristic that is objectively measured and evaluated as an indication of normal biological processes, pathogenic processes, or pharmacologic responses to a therapeutic intervention” [7]. Recent interest in biomarker discovery has been sparked by their successful clinical use in multiple areas of medicine [8], to aid in more objective and reliable diagnosis of a condition, or in predicting individual treatment response [9]. For example, in cancer research, the use of a stratification biomarker (HER2) significantly accelerated the development of an adjuvant immunotherapy for breast cancer, which reduced the risk of death by 33% in the subgroup of patients that were positive for the biomarker (cf. [10]).

In essence, a biomarker could be any characteristic or test outcome derived from genetic testing, biochemical assays, brain imaging scans, eye-tracking, or cognitive tests that make reliable predictions about an *individual*. It could be a continuous score that indicates clinical relevance from a certain cutoff point, a categorical score (e.g., the presence/absence of a particular genetic variant) that indicates the probability of a particular condition or subtype, or a composite derived from multiple indices.

### Biomarkers in homogeneous versus heterogeneous conditions

A *diagnostic biomarker* refers to a measurable characteristic that reflects the presence of a clinical umbrella condition and allows for definitive diagnosis [7]. Assuming that a condition is relatively homogeneous, such a marker should apply to all or most individuals with the condition, i.e., have high *sensitivity* (correctly classifying individuals as having the condition), high *specificity* (correctly classifying individuals as not having that particular condition), as well as high positive and negative predictive values. Currently, there are no established benchmarks for the statistical characteristics that *diagnostic biomarkers* have to fulfil. However, cutoffs of quantitative measures that allow classification [of the condition] with 80% sensitivity (correctly classifying individuals that are biomarker positive as having the condition) and 80% specificity (correctly classifying individuals that are biomarker negative as not having the condition (Cohen’s *d* of 1.66) is often considered as acceptable for diagnostic utility [11,12].

By contrast, assuming that a clinical condition is heterogeneous, a *stratification biomarker* refers to a measurable characteristic that can be used to identify more homogeneous biological subgroups within or across established diagnostic categories [4,7,13]. Thus, stratification biomarkers can be used to aid in the diagnosis of a subpopulation within a condition, to ascertain the likely development/progression of an individual with an umbrella condition and/or estimate the likely response to a given treatment/intervention [14] (see Table 1). These subgroups may be defined by particular participant characteristics (e.g., sex or age group). Alternatively, they may be defined by particular neurobiological characteristics (e.g., neurocognitive profile, brain atypicalities). In this case, we do not know how many subgroups may exist, how big they are, and what are the clinically relevant cutoffs.

**Table 1:** Biomarkers in homogeneous vs. heterogeneous conditions.

Biomarker definitions^$^	Homogeneous conditions	Heterogeneous conditions
**Diagnostic biomarker:** used to detect or confirm presence of a condition or to identify individuals with a subtype of the condition	Characteristic applies to most/all individuals with the umbrella condition. *Mean group difference with large effect size necessary*	a) Characteristic only applicable to a subset of individuals to aid in the diagnosis of a subtype of the condition. *Mean group difference with small effect size possible (or no effect if size of subgroup very small)* b) Different etiologies converge on a “final common pathway.” In this scenario, the characteristic may apply to most/all individuals with the umbrella condition; *mean group difference with large effect size necessary*
**Prognostic biomarker:** used to identify likelihood of a clinical event, recurrence, or progression in patients who have the condition.	Quantitative marker used to predict state of progression	Qualitative or quantitative marker used to predict different developmental trajectories/progression in individuals with the same umbrella condition
**Predictive biomarker:** used to identify individuals who are more likely than similar individuals without the biomarker to experience a favourable or unfavourable effect from exposure to a medical product	N/A. Predicts that a treatment/intervention only works in individuals with the given clinical condition—but not necessarily in others.	Marker predicts differential treatment response in individuals with/without biomarker positivity among umbrella condition
**Risk/likelihood biomarker**: indicates the potential for developing a condition in an individual who does not currently have […] the medical condition.	Marker predicts the likely development of the condition	Marker predicts the likely development of the condition in only a subtype

^$^Definitions abridged from [13].

Hence, an important step in biomarker discovery consists of moving beyond the focus on mean group differences and to establishing the frequency and severity of atypicalities on a given test or measure among individuals with a clinical condition.

Here, we first carried out simulations to examine how effect size, sample size, and the shape of distributions impact the likely utility of a biomarker. To exemplify this, we then applied these statistical criteria to evaluate the current state of biomarker discovery in one area of psychiatry where heterogeneity is well established—autism research. Other conditions with clinical and neurobiological heterogeneity include depression [15,16], ADHD [17], and even schizophrenia [18,19].

## Empirical data simulation

We generated 2 populations with varying sample sizes per group (20 and 100; see S1 Text for more details about the simulations). These sample sizes were chosen because approximately 15 to 30 participants per group has been the typical size of the majority of cognitive [20–22] or neuroimaging studies [23,24] in autism research (see S1 Table for a summary of representative meta-analyses, by domain).

### Identifying biomarkers in normal distributions: To what degree do 2 groups overlap at different effect sizes?

**Fig 1:**
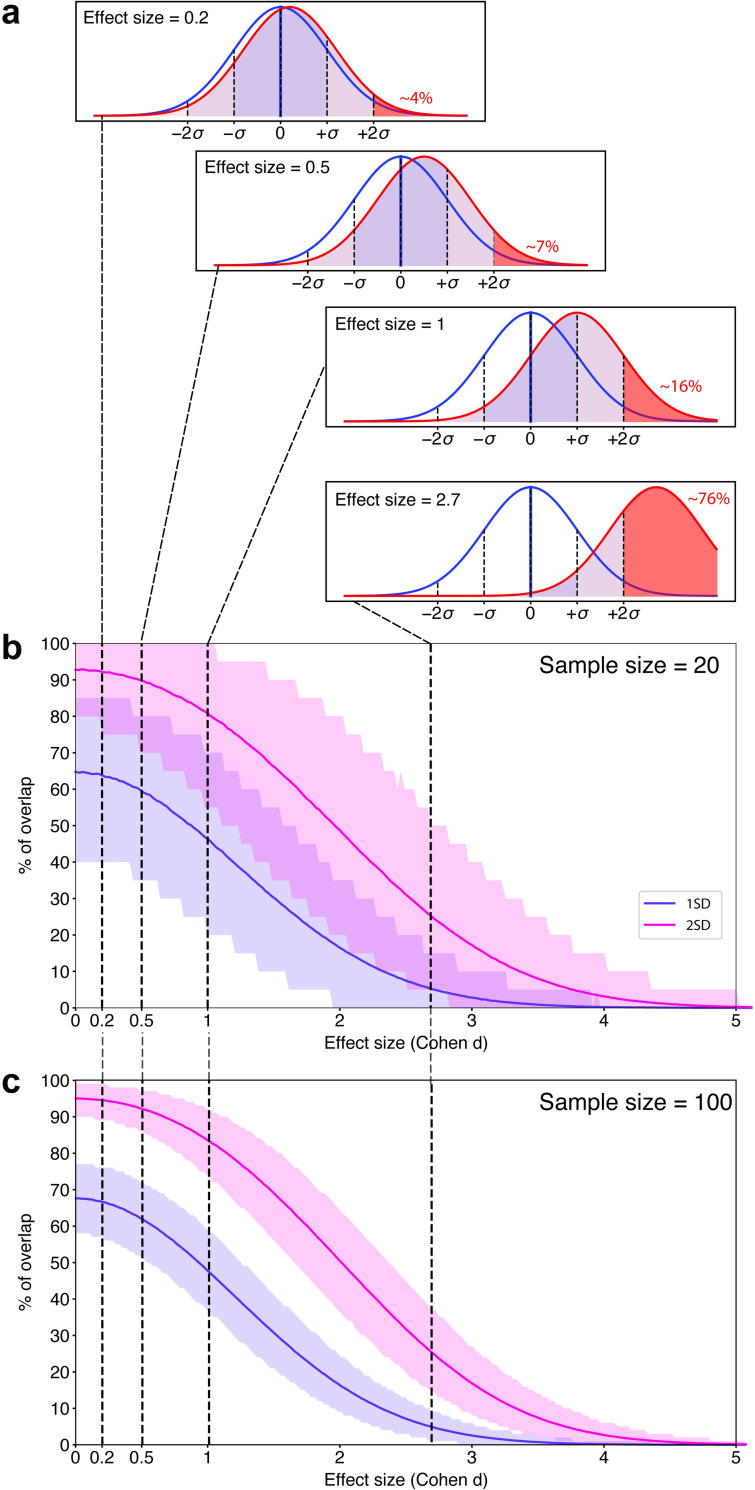
Simulations of the degree to which 2 groups overlap at different effect sizes. (a) Percentage of autistic individuals (red) falling within 1 SD and 2 SDs of the control (blue) distribution at effect size of *d* = 0.2, 0.5, 1, and 2.7. 0 = mean, σ = SD. Simulations based on 10,000 random draws assuming the same SD and absolute mean difference in the population. The red shaded area indicates the % of cases above 2 SDs. (b) Although sample size does not bias the effect size estimates themselves, it does substantially affect their *precision*, which is reflected in the width of the CI. The precision of effect size estimates with sample sizes of *N =* 20 and *N* = 100. Purple shading denotes CIs around 1 SD of the mean and red shading CIs around 2 SDs of the mean. For example, for a small effect size at Cohen’s *d* of 0.2, with *N* = 100 participants per group, between 60% and 75% of autistic people would fall within 1 SD of the control mean. With smaller samples of *N* = 20 per group, ranges grow to 40%–85% within 1 SD and to 75%–100% within 2 SDs. At Cohen’s *d* of 0.5, with *N* = 100 versus *N* = 20, 55%–71% versus 45%–80% of people with ASD would fall within 1 SD and between 89%–97% versus 85%–100% within 2 SDs of the TD mean, etc. Hence, with small sample sizes, the range of possible results is so wide that it is difficult to make accurate inferences of the frequency or severity of cases who have abnormalities on that measure from single studies. As recently noted, studies with small sample sizes (low power), paired with publication bias and file drawer effects as well as high sample variability (true heterogeneity within a condition), can lead a whole field to overestimate the magnitude of the true population effect [49,50]. ASD, autism spectrum disorder; CI, confidence interval; SD, standard deviation.

In our first set of simulations, we assumed that the test values of both the case and control groups are normally distributed (i.e., Gaussian). **Fig 1A** shows the average percentages of cases falling within 1 standard deviation (SD) (i.e., 68% around the control mean) and within 2 SDs (i.e., 95%). At Cohen’s *d* = 0.2 (which is considered a “small effect”), on average 67% of cases would fall within 1 SD of the control mean, at *d* = 0.5 (“medium effect”), on average 63% and at *d* = 1 (“large effect”), 48% of autistic people. For a normally distributed measure to have diagnostic utility as defined by 80% sensitivity and 80% specificity, Cohen’s *d* of 1.66 is required [12]. **S1 Fig** shows that in these distributions, still, 40% of cases have scores that fall within 1 SD of the control mean. To separate the scores of 75% of cases from the vast majority (>97.5%) of the control scores, very large effect sizes of Cohen’s *d* = 2.7 (AUC = 0.97) are needed [25].

However, a closer look at Fig 1 also indicates that there may be subgroups of individuals with nonoverlapping scores at medium or even small effect sizes. Note that even if a subgroup is relatively small (e.g., 5% to 10%), it could be clinically useful if for these individuals treatment response or developmental progress could be accurately predicted.

Fig 1B shows that assuming the same SD and absolute mean difference at the population level, the *precision* of effect size estimates depends on the sample size. For example, a study may report an effect size of *d* = 0.5. However, with a sample size of 20 per group, the percentage of cases who fall within 1 SDs of the control mean may actually vary between 35% to 80%. With a sample size of 100 per group, this range is reduced to 55% to 75%. This shows that with small samples, the range of estimates can be so large that they may be effectively meaningless.

### Identifying biomarkers in nonnormal distributions

**Fig 2:**
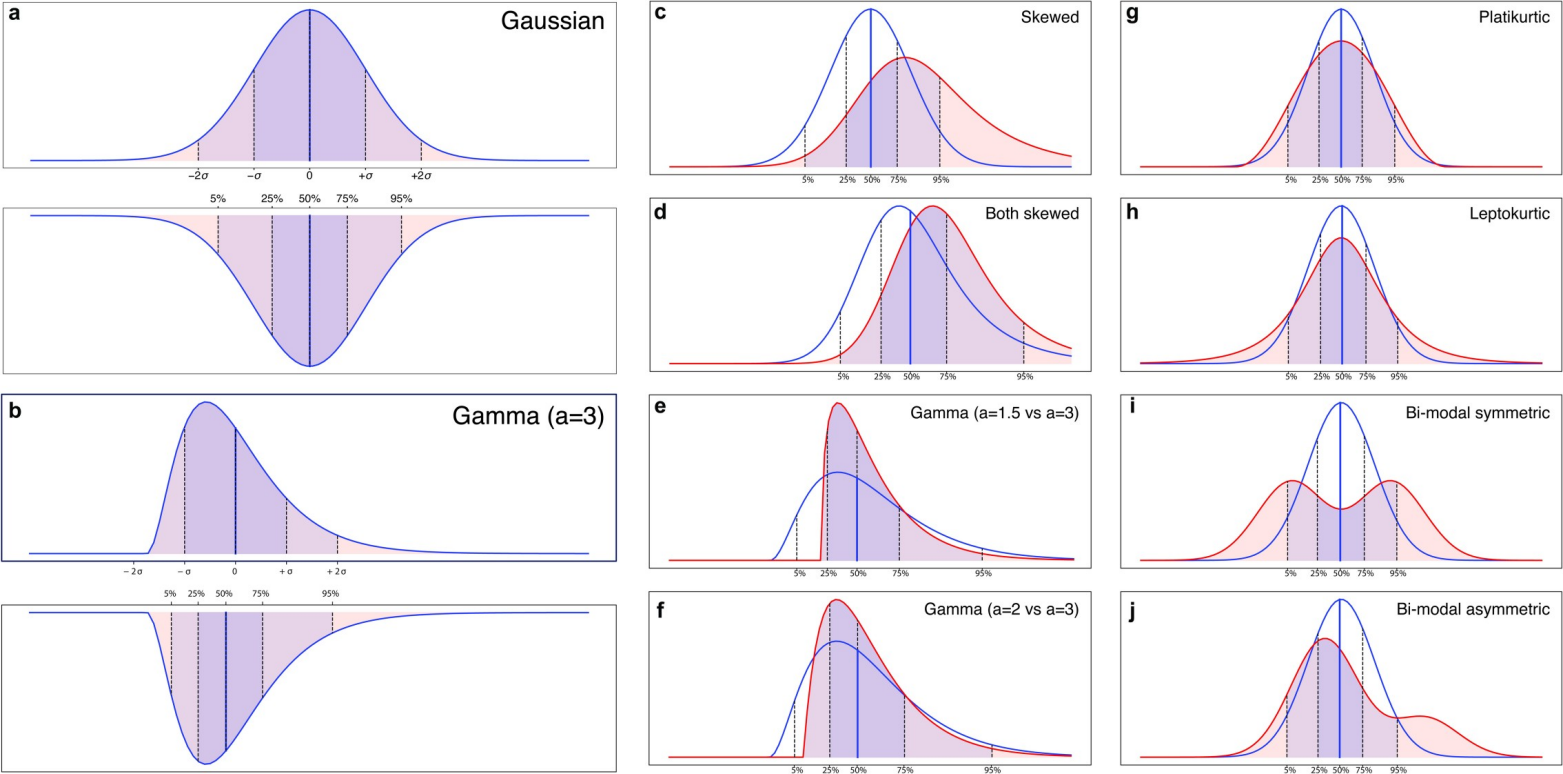
Simulation of how central tendencies and the shape of the distributions impact group overlap. Translating the central tendencies of mean and SD into median and interquartile ranges in (a) normal distribution and (b) skewed distribution. Illustration of group overlap when (c) case group is skewed but control group is normal, (d) both groups are skewed, (e) exponentially modified gamma distribution with strong skewness, and (f) with milder skewness, (g) platykurtosis, (h) leptokurtosis, (i), bimodal equal, (j) bimodal asymmetric. SD, standard deviation.

The next examples show that it is not sufficient to only focus on the shift in the central tendency between 2 groups; the shape of the distributions of both groups has to be considered as well (**Fig 2)**. Although differences in sample distributions may be minimised by careful experimental design, including selection of the comparison group, in clinical studies, quantitative measures have frequently been found to be nonnormally distributed [26–28]. Note that in nonnormal distributions, common reference values, such as means and SDs, as well as frequently used effect size measures, such as Cohen’s *d*, are not suitable [26]. **S2 Text and S2 Table** give a brief tutorial and a summary of how central tendencies of means and SDs can be translated into their nonparametric counterparts of median and percentiles. It is important to note that despite the increase of Type 1 error, most parametric statistical tests remain relatively robust when data depart from normality. However, despite such robustness in significance at the statistical level, the extent to which distributions diverge from normality can strongly impact effect sizes and thus could lead to misleading conclusions at the clinical level [29].

#### Skewness

When the data of only 1 group are skewed (Fig 2C), the number of individuals with atypical values increases relative to 2 normal distributions, yet this may not be obvious from the effect size value only. Skewness may indicate a larger subgroup with atypical (nonoverlapping) scores, while the majority of cases have scores in the same range as the comparison group. For instance, in the EU-AIMS LEAP cohort, we observed shifted developmental trajectories where the control group reached a ceiling effect in spatial working memory earlier than the autism group [30]. If the 2 groups are equally skewed, for example, due to a systematic bias in the measures, the degree of nonoverlap depends on the difference in the median (Fig 2D).

#### Platykurtosis

Fig 2E describes a distribution with negative kurtosis. They have lighter tails than a normal distribution, with more extreme scores at both ends. In psychiatric research, biologically plausible platykurtic distributions could be functional connectivity, where some cases might show hypoconnectivity and others hyperconnectivity relative to the control distribution.

The opposite case is *leptokurtosis*, with distributions presenting an excess positive kurtosis. Those distributions have fatter tails than normal. One scenario in which this could happen in psychiatric research is when the control group’s “true” distribution on a test or measure is normal, but participants are sampled from a specific subpopulation, such as University students, who may display a narrower range of scores. If 1 group is platykurtic (or leptokurtic), we could find the seemingly paradoxical situation where the 2 group means and medians are identical, i.e., statistically no effect was obtained—and yet 2 subgroups exist with nonoverlapping values. In other words, we can have a stratification biomarker indicating biologically plausible subgroups in the absence of a significant mean group difference.

#### Bimodality/multimodality

Similarly, bimodal distributions are more likely to indicate a stratification biomarker than diagnostic marker, as they are a continuous probability distribution with 2 different modes. A simple bimodal distribution could be a mixture of 2 normal distributions with different means but the same variance (Fig 2F). Similar to other situations discussed above, one can measure how far each individual deviates from the median of the nearest relevant mixture distribution.

If the weights are not equal, the distribution could still be bimodal but with peaks of different heights (Fig 2G**)**. In autism research, this is exemplified by hyperserotonemia that has been found in approximately 30% of autistic individuals [31,32].

**S2 Table** gives examples of the percentage of individuals within 68% and 95% of the median values as a function of (i) differences in the group medians; and (ii) specific levels of skewness or distribution width.

### Comparison of effect sizes reported in meta-analyses of different areas of autism research

Next, we investigated whether mean group differences obtained across the most influential areas of autism research meet our theoretical considerations for diagnostic biomarkers. For illustrative purposes, we selected 1 representative published meta-analysis per domain and compiled average effect sizes for theory of mind [21], executive function [22], emotion recognition [20], eye-tracking [33], EEG of mismatch negativity [23], and N170 [34], functional MRI of reward processing [24], structural MRI [35], and genetics [36].

**Fig 3:**
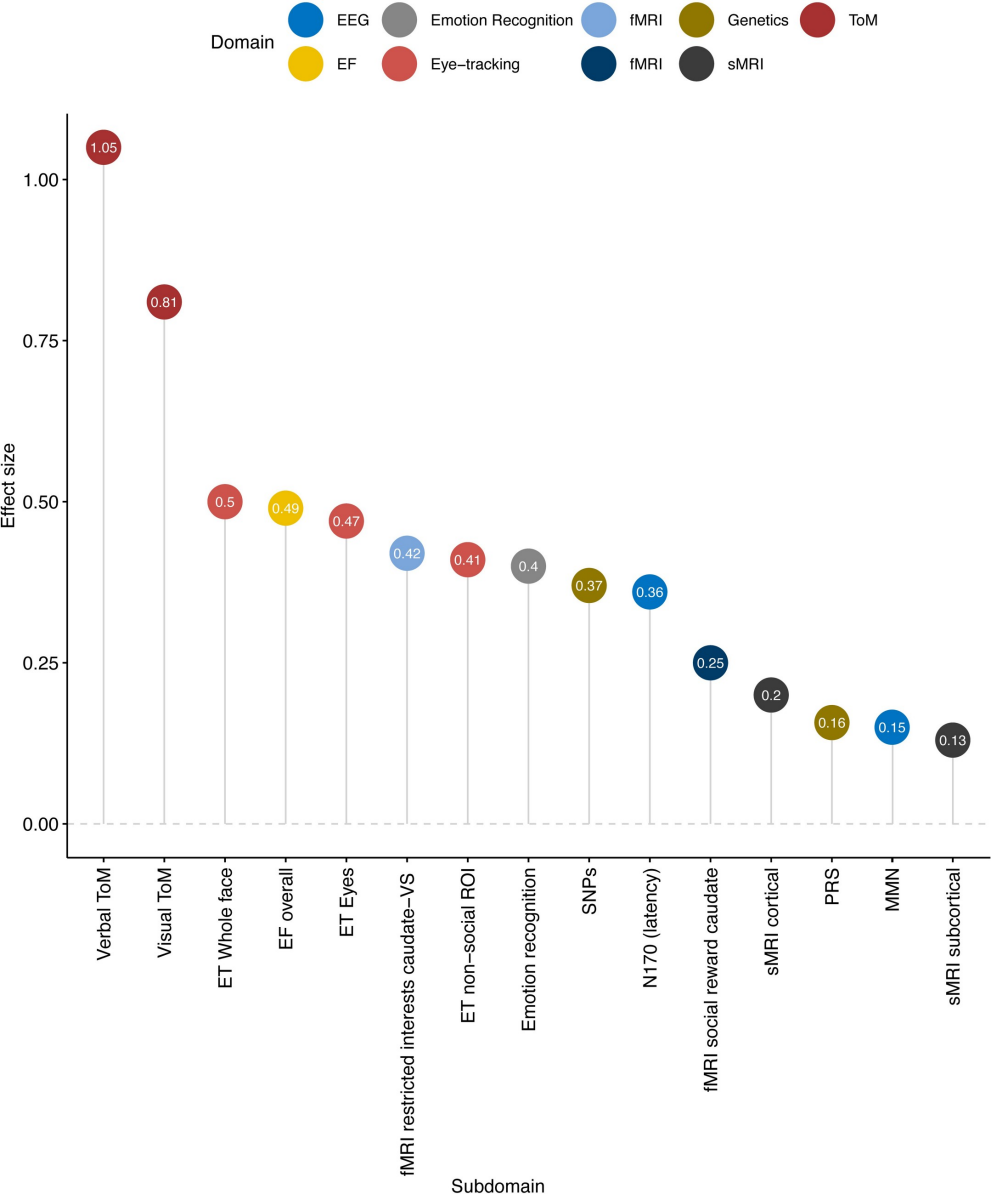
Average effect sizes of meta-analyses per modality. The distributions of the original data included in the meta-analyses were often not reported. This is exemplified in S1 Table where we checked information on the data distributions of the 49 original papers included in a review of emotion recognition [20]. However, in the majority of papers, parametric statistics were employed, which may be taken as indicating normal distribution. EF, executive function; ET, eye-tracking; fMRI, functional MRI; MMN, mismatch negativity; PRS, polygenic risk score; ROI, region of interest; sMRI, structural MRI; SNP, single-nucleotide polymorphism; ToM, theory of mind.

As shown in Fig 3, the largest effect sizes were found in theory of mind (with *d*’s from 0.8 to 1.1) [37]. Moderate effect sizes were found in meta-analyses of emotion recognition *d* = 0.8 [20], across different aspects of executive function (*d* = 0.45 to *d* = 0.55), in eye-tracking studies (*d* = 0.4 to 0.5) [22,33], and EEG studies of the N170 event-related potential response to faces (increased latency *d* = 0.36). One of the first meta-analysis of fMRI studies that did report effect sizes showed that in the area of reward processing, effect sizes ranged from *d* = 0.025 to 0.42 [24]. A recent mega-analysis of brain anatomy reported effect sizes ranging from Cohen’s *d* = 0.13 for subcortical volumes to *d* = 0.21 for cortical thickness [35]. Finally, GWAS results from a recent meta-analysis [36] yielded small effect sizes *d* = 0.06 with single-nucleotide polymorphisms (SNPs) passing corrected *p*-value for association to *d* = 0.37 with those passing nominal threshold of (i.e., *p* < 0.05). Effect sizes of polygenic risk score (PRS), which combines the signal across the SNPs [38], translated to a Cohen’s *d* of 0.16 [36]. Notice that with such small effect sizes, genetics studies often employ much larger sample sizes (see **S2 Fig** for additional simulations).

**S3 Table** shows for the area of emotion recognition that original papers often do not report how the data were distributed. However, if we take the authors’ use of parametric statistics as an index that the data were normally distributed and compare these findings to our simulations we find that—across the different areas of autism research and despite significant mean group differences—approximately 48% to 68% of autistic individuals would fall within 1 SD of the typical range; i.e., they do not have a deficit or atypicality in a statistical sense, or of likely clinical relevance. This conclusion drastically contrasts the way mean group differences are often reported in the autism literature where the “on average” is very frequently omitted. To quantify the extent of this practice, we carried out a PubMed search with the search terms “autism,” [domain], e.g., “eye-tracking,” “structural MRI,” “EEG,” with or without the additional terms “on average” in the abstract. Table 2 shows that across domains, only between 1.8% and 4.6% of studies used the term “mean group difference” or “on average” when summarising their findings. Of studies that investigated mean group differences as potential biomarkers, these were up to 5.5%. Instead, common interpretations of findings included phrases, such as, “we demonstrate that people with autism have reduced [X], “[X] is characteristic for autism,” or even “findings suggest that [X] may be a biomarker for autism.” (References were here deliberately omitted so not to single out individual studies/authors). This way of reporting can be misleading as it tacitly implies that the group level difference generalises to all individuals in that group.

**Table 2:** Use of terms “on average” and/or “biomarker” in published papers using PubMed search, across domains.

	Autism	Autism + on average*	Autism + biomarker	Autism + biomarker + on average
Eye-tracking	567	26	43	0
Structural MRI	1,058	41	99	5
fMRI	3,284	118	229	13
EEG	1,807	86	159	8
Executive function	896	37	19	0
Emotion recognition	869	23	15	0
Theory of mind	1,169	18	7	0
Genetics	15,524	281	979	22

*The figures in this column are likely an overestimation as they include the use of “on average” in contexts other than referring to group differences, for example, to characterise participants (e.g., “the ASD group had IQ on average between X and Y”).

The meta-analyses were selected based on the following search criteria:

((autism[Title/Abstract]) AND (meta analysis[Title/Abstract])) AND [“DOMAIN”/Title/Abstract]. Searches were repeated iteratively for the domains structural MRI, emotion recognition, theory of mind, eye-tracking, fMRI, EEG, and genetics, each for the past 10 years. For illustrative purposes, we then selected 1 meta-analysis per domain based on the following criteria: number of citations, journal impact, and comprehensiveness of the meta-analysis.

### Using case–control comparisons to explore the potential value of a measure as a stratification biomarker

Even if mean group differences with medium to even large effects may often not be indicative of a diagnostic biomarker, they may still provide pointers for possible subgroups (stratification biomarker) with the given characteristic. Box 1 provides a checklist of some concrete steps that may help interested researchers to interrogate their data from a biomarker perspective. Several of our examples highlight that identification of stratification biomarkers (subgroups) within or even across diagnostic disorders requires considerably larger sample sizes than those that have previously been typically carried out. Sample size not only affects *p*-values and the precision of the effect size, small samples may make it difficult to detect a potentially small but clinically relevant subgroups (e.g., 10% of a disorder). Recently, across psychiatry, larger consortia have been funded that address this issue.

**Box 1. Checklist for researchers**

- Check if data are normally distributed or not using Kolmogorov–Smirnoff or Shapiro–Wilk tests; graphical methods such as simple histograms or robust graphical methods help to visually understand the distribution of the data. Rule of thumb: if the standard deviation (SD) exceeds half of the mean value, the distribution of the data is likely nonnormal.
- Use the shape of the distribution as a starting point to evaluate data points in addition to means or medians. For example, Bayesian information criterion (BIC) and Akaike information criterion (AIC) can be used to decide whether a distribution may be best described as “skewed” versus harbouring a mixture model.
- Reference values to estimate the frequency and severity of atypicalities: Although means and SDs can be appropriately used when data are normally distributed (or can be transformed to normal distributions), we recommend using median and percentiles (e.g., interquartile ranges, SD from the median) in order to facilitate the comparison of results across different data distributions.
- Always provide confidence intervals irrespective of sample size so that readers can judge whether the estimate is sufficiently precise for their purposes. If the data set is small, be aware of limited “precision” of effect size estimates.
- Preanalytic validation: We recommend carrying out test–retest reliability for any new measures or measures for which such information is not available. While no universally acceptable values are available, several textbooks recommend r > 0.70. Test variability (and potentially known moderating factors, e.g., fatigue, time of day) should be included in the interpretation of scores. Standardisation of instructions, standard operating procedures (SOPs), acquisition, and preprocessing parameters are essential to ensure comparability between researchers, clinicians, and laboratories. Measures should be optimised and key dependent variables specified.
- Clinical validation and/or validation against another level of explanation: Relate single measure to clinical outcome, define cutoff for clinical relevance accordingly.
- Increase scientific knowledge by facilitating replication, script, and data sharing: Seek to replicate findings of percentages and relationship to external variables in an independent data set.
- Actively deposit your data set in open data repositories (e.g., Zenodo, Open Science Framework) and share your task paradigm and scripts with other colleagues to enable data pooling and replication.

In addition to analytic validation of the candidate biomarker, we also need to demonstrate its clinical relevance and determine clinically relevant cutoffs, such that individuals with (different degrees of) biomarker positivity differ from those with biomarker negativity in terms of specific clinical features. This biomarker-clinical phenotype relationship could be linear or nonlinear such that the atypicality only becomes clinically relevant from a certain degree or “tipping point” [39]. There are currently no general benchmarks for the accuracy of biomarkers, as, for example, the required sensitivity/specificity of a subgroup may depend on the particular context of use of the biomarker (e.g., treatment prediction, prognosis) and associated cost-benefits (e.g., financial cost of treatment, side effects, etc.) [40].

As a limitation of our illustrations, it should be noted that we only considered biomarkers based on a single continuous measure because of the historical prevalence of univariate approaches in neuropsychiatry. In the context of precision medicine, a host of multivariate methods have been recently applied to high-dimensional data sets. Given the complexity of processes and mechanisms underpinning most psychiatric conditions, it is likely that they cannot be captured by 1 biomarker. Moreover, independent single features may have small effect size, yet a group of such features considered in a multivariate fashion might effectively have a “high effect size”—the so-called Lewontin’s fallacy [41]. Hence, multiple scores may be combined through predictive pattern-learning algorithms to identify subtypes [42–44] (for review, see [45]).

## Conclusions

Our systematic simulations show that the statistical significance of mean group differences alone is a poor indicator of the likely utility of a measure or test as a (diagnostic) biomarker. Although statisticians are well aware of these basic principles [6,26,46–48], a review of these principles is timely since they are still too often ignored or misunderstood. By using autism research as an example, we have shown that mean group differences with moderate to even large effect sizes (Cohen’s *d*s from 0.5 to 1.0) are not indicative of a “diagnostic biomarker.” Instead, significant mean group differences with moderate effect sizes often indicate that many, if not the majority of cases do *not* have an impairment or atypicality on that measure. However, 1 (skewness) or 2 (platykurtic, bimodal) subgroups may exist despite small or nonsignificant overall effects. The same principles apply to other areas of neuropsychiatry or medicine more broadly. We outline some specific steps to further explore these findings as potential stratification biomarkers and surmise that similar considerations may be applicable for other areas of neuropsychiatry.

## Supporting information

S1 Table Example meta-analyses across the most influential areas of autism research. Authors, effect size, total, and average sample sizes per group. (DOCX)

S2 Table Examples of effect sizes and reference boundaries for different types of distributions. (DOCX)

S3 Table Author’s reporting of the distribution of data and the use of parametric vs. nonparametric statistics, using the meta-analysis of emotion recognition (Uljarevic and Hamilton, 2013) as an example. (DOCX)

S1 Fig Relationship between ROC curve and distribution overlap for an effect size of *d* = 1.66. The coloured circles indicate key thresholds at half the control group distribution (blue; i.e., 0.5 specificity), at the best separation between the groups (purple; 80% sensitivity, 80% specificity), and at half of the patient distribution (red; i.e., 50% sensitivity). Inspired by the ROC curve interactive demonstration http://arogozhnikov.github.io/2015/10/05/roc-curve.html. AUC, area under the curve; ROC curve, receiver operating characteristic curve.
(TIF)

S2 Fig Complementary simulations for the quantification of the impact of sample size on the reliability of effect size estimates. (a) Case of an effect size of 1, corresponding to approximately the largest observed in the meta-analysis. (b) Case of an effect size of 0.16, corresponding to the one associated with PRS. For the latter, the point estimate is indicated by the vertical black line, while the colour lines represent the probability distribution of the effect size estimates across 10,000 simulations and with sample sizes of the PGC (18,381 individuals with ASD and 27,969 controls), EU-AIMS (500 ASD and 500 controls), and those used for Fig 1, i.e., *N =* 20 and 100. ASD, autism spectrum disorder; PRS, polygenic risk score.
(TIF)

S1 Text Scientific computing and empirical simulations. Python (Python Software Foundation; https://www.python.org/) was selected as the scientific computing engine. Capitalising on its open-source ecosystem helps enhance replicability, reusability, and provenance tracking. The Numpy (van der Walt and colleagues, 2011), Scipy (Virtanen and colleagues, 2020), and Matplotlib (Hunter, 2007) packages were used to generate all numerical simulations. Scripts that reproduce the results of the present study are readily accessible and open for reuse (https://gist.github.com/deep-introspection/4280aeee34a0f1ab4491a386adcd5dad/). We generated 2 populations with varying sample sizes per group (20 and 100).
(DOCX)

S2 Text Translating means and SDs into nonparametric equivalents to assess the frequency and severity of atypicalities in nonnormal distributions. Here, we review how we can calculate the frequency and severity of atypicalities when the data of one or both groups are nonnormally distributed. The degree to which 2 distributions overlap depends both on the differences between the central tendencies *and* the shape of the distributions. Previously, we used the mean and SD of the comparison group as reference points to estimate how far a given individual diverges from the typical range. Cohen’s *d* was used as an index of the magnitude of the group separation. In normal distributions, the mean and median are the same and represent the most typical value in the data set. However, in skewed or gamma distributions, the mean is dragged more into the direction of the skew (“longer tail”) than is the median. In many instances of skewed distributions, the median is therefore the more appropriate central tendency as it characterises where the majority of individuals scored. There are a number of effect size measures available that are more “robust” to skewness, such as the scaled/unscaled robust *d* or the common language effect size (Li and colleagues, 2016). However, our primary interest is not in an index of the magnitude of the effect per se, but in finding a way to ascertain the frequency and severity of atypicalities on a test/measure in a clinical group. This requires us to move to nonparametric statistics. Therefore, we translated the central tendencies of means and SDs into their nonparametric counterparts of median and percentiles. The IQR is calculated by dividing the data set into 4 equal portions and refers to the “middle 50%,” i.e., the range between the 25th percentile (Q1) and the 75% percentile (Q3). The 50th percentile or (Q2) is then the same as the median. The IQR is somewhat narrower than 1 SD. The equivalent to 1 SD (68% of values) of the mean would be 68% around the median. This now provides us with a universal way to express frequencies and severities across different types of distributions. IQR, interquartile range; SD, standard deviation.
(DOCX)
