# Supplementary material for: The meaning of significant mean group differences for biomarker discovery
Source: PLoS Comput Biol. 2021 Nov 18;17(11):e1009477. doi: 10.1371/journal.pcbi.1009477 (PMC8601419; doi:10.1371/journal.pcbi.1009477)
Supplement: S1 Table — (DOCX) [file pcbi.1009477.s001.docx]

**S1 Table. Example meta-analyses across the most influential areas of autism research. Authors, effect size, total and average sample sizes per group**

| Author | Domain | Subdomain | ES | 95% CI | ASD Sample | TD sample | overall sample | average N ASD | average N TD |
| --- | --- | --- | --- | --- | --- | --- | --- | --- | --- |
| van Rooij (2018) [1] | sMRI | sMRI cortical | 0.2 | Na* | 1571 | 1651 | 3222 | 32.2 (7-102) | 32.1 (0-154) |
|  | sMRI | sMRI subcortical | 0.13 |  | 1571 | 1651 | 3222 | 32.2 (7-102) | 32.1 (0-154) |
| Uljarevic & Hamilton (2013) [2] | Emotion Recognition | Emotion recognition | 0.8 | Na* | 980 | 1282 | 2262 | 17.4 (5-90) | 17.1 (5-55) |
| Chung et al. (2014) [3] | ToM | verbal ToM | 1.05 | 0.6-1.5 | 230 | 192 | 422 | 21.1 (6-61) | 19.7 (10-41) |
|  | ToM | visual ToM | 0.81 | 0.48-1.14 | 246 | 249 | 495 | 21.1 (6-61) | 19.7 (10-41) |
| Demetriou et al. (2017) [4] | EF | EF overall | 0.49 | 0.44-0.55 | 6816 | 7265 | 14081 | 29.2 (7-149) | 31.2 (7-155) |
| Schwartz et al (2018) [5] | EEG | MMN | 0.15 | -0.34 | 307 | 280 | 587 | 16.2 (7-51) | 15.2 (7-34) |
| Kang et al (2018) [6] | EEG | N170 (latency) | 0.36 | 0.06-0.67 | 374 | 359 | 733 | 18.7 (8-43) | 18.1 (9-40) |
| Frazier et al. (2016) [7] | Eye-tracking | ET Eyes | 0.47 | Na* | 2199 | 2418 | 4617 | n/a | n/a |
|  | Eye-tracking | ET Whole face | 0.5 | Na* | 2199 | 2418 | 4617 | n/a | n/a |
|  | Eye-tracking | ET non-social ROI | 0.41 | Na* | 2199 | 2418 | 4617 | n/a | n/a |
| Clements et al. (2018) [8] | fMRI | social reward caudate | 0.25 | -0.5 | 259 | 246 | 505 | 19.9 (10-39) | 18.9 (10-29) |
|  | fMRI | restricted interests caudate-VS | 0.42 | 0.07-.78 | 259 | 246 | 505 | 19.9 (10-39) | 18.9 (10-29) |
|  |  |  |  |  |  |  |  |  |  |
|  |  |  |  |  |  |  |  |  |  |
| Grove et al, 2019 [9] | Genetics | SNPs association | 0.37 | 1.3-1.36 | 18381 | 27969 | 46350 | n/a | n/a |

The meta-analyses were selected based on the following search criteria:

((autism[Title/Abstract]) AND (meta analysis[Title/Abstract])) AND [“DOMAIN”/ Title/ Abstract]. Searches were repeated iteratively for the domains structural MRI, emotion recognition, theory of mind, eye-tracking, fMRI, EEG and genetics, each for the past 10 years. For illustrative purposes, we then selected one meta-analysis per domain based on the following criteria: number of citations, journal impact, and comprehensiveness of the meta-analysis.

*Na= not available

References:

1. van Rooij D, Anagnostou E, Arango C, Auzias G, Behrmann M, Busatto GF, et al. Cortical and Subcortical Brain Morphometry Differences Between Patients With Autism Spectrum Disorder and Healthy Individuals Across the Lifespan: Results From the ENIGMA ASD Working Group. Am J Psychiatry. 2017; appi.ajp.2017.17010100. doi:10.1176/appi.ajp.2017.17010100

2. Uljarevic M, Hamilton A. Recognition of emotions in autism: a formal meta-analysis. J Autism Dev Disord. 2013;43: 1517–1526. doi:10.1007/s10803-012-1695-5

3. Chung YS, Barch D, Strube M. A Meta-Analysis of Mentalizing Impairments in Adults With Schizophrenia and Autism Spectrum Disorder. Schizophr Bull. 2014;40: 602–616. doi:10.1093/schbul/sbt048

4. Demetriou EA, Lampit A, Quintana DS, Naismith SL, Song YJC, Pye JE, et al. Autism spectrum disorders: a meta-analysis of executive function. Mol Psychiatry. 2018;23: 1198–1204. doi:10.1038/mp.2017.75

5. Schwartz S, Shinn-Cunningham B, Tager-Flusberg H. Meta-analysis and systematic review of the literature characterizing auditory mismatch negativity in individuals with autism. Neurosci Biobehav Rev. 2018;87: 106–117. doi:10.1016/j.neubiorev.2018.01.008

6. Kang E, Keifer CM, Levy EJ, Foss-Feig JH, McPartland JC, Lerner MD. Atypicality of the N170 Event-Related Potential in Autism Spectrum Disorder: A Meta-analysis. Biol Psychiatry Cogn Neurosci Neuroimaging. 2018;3: 657–666. doi:10.1016/j.bpsc.2017.11.003

7. Frazier TW, Strauss M, Klingemier EW, Zetzer EE, Hardan AY, Eng C, et al. A Meta-Analysis of Gaze Differences to Social and Nonsocial Information Between Individuals With and Without Autism. J Am Acad Child Adolesc Psychiatry. 2017;56: 546–555. doi:10.1016/j.jaac.2017.05.005

8. Clements CC, Zoltowski AR, Yankowitz LD, Yerys BE, Schultz RT, Herrington JD. Evaluation of the Social Motivation Hypothesis of Autism: A Systematic Review and Meta-analysis. JAMA Psychiatry. 2018;75: 797. doi:10.1001/jamapsychiatry.2018.1100

9. Grove J, Ripke S, Als TD, Mattheisen M, Walters RK, Won H, et al. Identification of common genetic risk variants for autism spectrum disorder. Nat Genet. 2019;51: 431. doi:10.1038/s41588-019-0344-8
