# Supplementary material for: The meaning of significant mean group differences for biomarker discovery
Source: PLoS Comput Biol. 2021 Nov 18;17(11):e1009477. doi: 10.1371/journal.pcbi.1009477 (PMC8601419; doi:10.1371/journal.pcbi.1009477)
Supplement: S2 Table — (DOCX) [file pcbi.1009477.s002.docx]

**S2 Table. Examples of effect sizes and reference boundaries for different types of distributions**

|  | Effect size measures | | | | Reference boundaries | | | | |
| --- | --- | --- | --- | --- | --- | --- | --- | --- | --- |
| Distribution | *d* | Robust D_r_ | Common language EF A_w_ | AUC | IQR  (25-50%) | (15.9%-84.1%) | (2.3%-97.7%) | Below 1SD (Proportion) | Below 2SDs (Proportion) |
| Normal  *d*=0.2 | 0.20 | 0.20 | 0.56 | 0.53 | 0.49 | 0.67 | 0.95 | 0.21 | 0.04 |
| Normal  *d*=0.5 | 0.50 | 0.50 | 0.64 | 0.57 | 0.45 | 0.62 | 0.93 | 0.31 | 0.07 |
| Normal  *d*=1 | 1.00 | 1.00 | 0.76 | 0.63 | 0.33 | 0.48 | 0.84 | 0.50 | 0.16 |
| Normal  *d*=1.5 | 1.50 | 1.50 | 0.86 | 0.69 | 0.19 | 0.30 | 0.69 | 0.69 | 0.31 |
| Normal  *d*=2 | 2.00 | 2.00 | 0.92 | 0.75 | 0.09 | 0.16 | 0.50 | 0.84 | 0.50 |
| Normal  *d*=2.7 | 2.70 | 2.70 | 0.97 | 0.82 | 0.02 | 0.04 | 0.24 | 0.96 | 0.76 |
| One Skewed  *d*=1 | 1.00 | 0.95 | 0.76 | 0.64 | 0.28 | 0.41 | 0.69 | 0.55 | 0.31 |
| Both Skewed  *d*=1 | 0.58 | 0.65 | 0.68 | 0.74 | 0.46 | 0.65 | 0.95 | 0.28 | 0.08 |
| Both Gamma  (a=3 vs a=1.5) | 0.00 | 0.01 | 0.53 | 0.42 | 0.74 | 0.90 | 0.99 | 0.09 | 0.02 |
| Both Gamma  (a=3 vs a=2) | 0.00 | 0.00 | 0.52 | 0.43 | 0.62 | 0.82 | 0.99 | 0.11 | 0.03 |
| Platikurtic | -0.07 | -0.03 | 0.51 | 0.51 | 0.41 | 0.58 | 0.92 | 0.18 | 0.03 |
| Leptokurtic | 0.00 | 0.00 | 0.50 | 0.50 | 0.40 | 0.55 | 0.83 | 0.22 | 0.09 |
| Bimodal  Symmetric | 0.00 | 0.00 | 0.50 | 0.50 | 0.19 | 0.30 | 0.69 | 0.35 | 0.15 |
| Bimodal  Asymmetric | 0.19 | 0.03 | 0.51 | 0.53 | 0.34 | 0.49 | 0.77 | 0.28 | 0.18 |

*D=*Cohen’s *d,* standardized mean difference

Robust effect size measures:

d_r_=unscaled robust d

A_w_= non-parametric estimator for common-language effect size

AUC= Area under the Curve

IQR= Interquartile range
