# Supplementary material for: The meaning of significant mean group differences for biomarker discovery
Source: PLoS Comput Biol. 2021 Nov 18;17(11):e1009477. doi: 10.1371/journal.pcbi.1009477 (PMC8601419; doi:10.1371/journal.pcbi.1009477)
Supplement: S3 Table — (DOCX) [file pcbi.1009477.s003.docx]

**S3 Table. Author’s reporting of the distribution of data and the use of parametric vs. non-parametric statistics, using the meta-analysis of emotion recognition [1] as an example**

| \| Study \| Did authors report the distribution of their data before analysis? \| if **NO**, did they use parametric or non-parametric stats? \| \| \| if **YES**, what type of distribution do they report having? (eg. gamma, bi-modal, mixture, poisson, etc.) \| \| --- \| --- \| --- \| --- \| --- \| --- \| \| Atkinson, A. P. (2009). Impaired recognition of emotions from body movements is associated with elevated motion coherence thresholds in autism spectrum disorders. Neuropsychologia, 47(13), 30233029. \| No \| Parametric \| \| \|  \| \| Baron-cohen, S., Spitz, A., & Cross, P. (1993). Cognition & Emotion, 7(6), 507-516. \| Not available \|  \|  \| \| \| \| Bolte, S., & Poustka, F. (2003). The recognition of facial affect in autistic and schizophrenic subjects and their first-degree relatives. Psychological medicine,33(5), 907-915. \| No \| Parametric \| \|  \| \| \| Boraston, Z., Blakemore, S. J., Chilvers, R., & Skuse, D. (2007). Impaired sadness recognition is linked to social interaction deficit in autism.Neuropsychologia, 45(7), 1501-1510. \| No \| Parametric \| \|  \| \| \| Braverman, M., Fein, D., Lucci, D., & Waterhouse, L. (1989). Affect comprehension in children with pervasive developmental disorders. Journal of autism and developmental disorders, 19(2), 301-316. \| No \| Parametric \| \|  \| \| \| *Buitelaar, J. K., Van der Wees, M., Swaab-Barneveld, H. A. N. N. A., & Van Der Gaag, R. J. (1999). Theory of mind and emotion-recognition functioning in autistic spectrum disorders and in psychiatric control and normal children.Development and Psychopathology, 11(1), 39-58. \| not available \|  \|  \| \| \| \| Castelli, F. (2005). Understanding emotions from standardized facial expressions in autism and normal development. Autism, 9(4), 428-449. (experiment 2) \| No \| non-parametric \| \| \| \| \| Celani, G., Battacchi, M. W., & Arcidiacono, L. (1999). The understanding of the emotional meaning of facial expressions in people with autism. Journal of autism and developmental disorders, 29(1), 5766. \| No \| Parametric \| \|  \| \| \| Clark, T. F., Winkielman, P., & McIntosh, D. N. (2008). Autism and the extraction of emotion from briefly presented facial expressions: Stumbling at the first step of empathy. Emotion, 8(6), 803. \| not available \|  \| \|  \| \| \| Corden, B., Chilvers, R., & Skuse, D. (2008). Avoidance of emotionally arousing stimuli predicts social–perceptual impairment in Asperger's syndrome.Neuropsychologia, 46(1), 137-147. \| not available \|  \| \|  \| \| \| Da Fonseca, D., Santos, A., Bastard-Rosset, D., Rondan, C., Poinso, F., & Deruelle, C. (2009). Can children with autistic spectrum disorders extract emotions out of contextual cues?. Research in Autism Spectrum Disorders,3(1), 50-56. \| not available \|  \| \|  \| \| \| *Davies, S., Bishop, D., Manstead, A. S., & Tantam, D. (1994). Face perception in children with autism and Asperger's syndrome. Journal of Child Psychology and Psychiatry, 35(6), 1033-1057. (experiement 2) \| No \| Parametric \| \|  \| \| \| *Deruelle, C., Rondan, C., Gepner, B., & Tardif, C. (2004). Spatial frequency and face processing in children with autism and Asperger syndrome. Journal of autism and developmental disorders, 34(2), 199-210. \| No \| Parametric \| \|  \| \| \| Downs, A., & Smith, T. (2004). Emotional understanding, cooperation, and social behavior in high-functioning children with autism. Journal of autism and developmental disorders, 34(6), 625-635. \| No \| Parametric \| \|  \| \| \| *Dyck, M. J., Ferguson, K., & Shochet, I. M. (2001). Do autism spectrum disorders differ from each other and from non-spectrum disorders on emotion recognition tests?. European child & adolescent psychiatry, 10(2), 105-116. \| No \| Parametric \| \|  \| \| \| Dziobek, I., Fleck, S., Rogers, K., Wolf, O. T., & Convit, A. (2006). The ‘amygdala theory of autism’revisited: Linking structure to behavior.Neuropsychologia, 44(10), 1891-1899. \| fMRI study \|  \| \|  \| \| \| *Fein, D., Lueci, D., Braverman, M., & Waterhouse, L. (1992). Comprehension of affect in context in children with pervasive developmental disorders. Journal of Child Psychology and Psychiatry, 33(7), 1157-1162. \| No \| Parametric \| \|  \| \| \| *Gepner, B., Deruelle, C., & Grynfeltt, S. (2001). Motion and emotion: A novel approach to the study of face processing by young autistic children. Journal of autism and developmental disorders, 31(1), 3745. \| No \| Parametric \| \|  \| \| \| Gepner, B., de Gelder, B., & de Schonen, S. (1996). Face processing in autistics: Evidence for a generalised deficit?. Child Neuropsychology, 2(2), 123-139. \| No \| Parametric \| \|  \| \| \| Grossman, R. B., & Tager-Flusberg, H. (2008). Reading faces for information about words and emotions in adolescents with autism. Research in autism spectrum disorders, 2(4), 681-695. \| No \| Parametric \| \|  \| \| \| Grossman, J. B., Klin, A., Carter, A. S., & Volkmar, F. R. (2000). Verbal bias in recognition of facial emotions in children with Asperger syndrome. Journal of Child Psychology and Psychiatry, 41(3), 369-379. \| No \| Parametric \| \|  \| \| \| Hadjikhani, N., Joseph, R. M., Manoach, D. S., Naik, P., Snyder, J., Dominick, K., ... & De Gelder, B. (2009). Body expressions of emotion do not trigger fear contagion in autism spectrum disorder. Social cognitive and affective neuroscience, 4(1), 70-78. \| No \| Parametric \| \|  \| \| \| Hobson, R. P. (1986). The autistic child's appraisal of expressions of emotion.Journal of Child Psychology and Psychiatry, 27(3), 321-342. \| Yes \| Non-parametric \| \| Bi-modal for autistic children, describe ceiling effect for non-autistic children \| \| \| Hubert, B., Wicker, B., Moore, D. G., Monfardini, E., Duverger, H., Fonseca, D. D., & Deruelle, C. (2007). Brief report: recognition of emotional and non-emotional biological motion in individuals with autistic spectrum disorders.Journal of autism and developmental disorders, 37(7), 1386-1392. \| No \| Parametric \| \|  \| \| \| Humphreys, K., Minshew, N., Leonard, G. L., & Behrmann, M. (2007). A fine-grained analysis of facial expression processing in high-functioning adults with autism. Neuropsychologia, 45(4), 685-695. \| No \| Parametric \| \| \| \| \| Jones, C. R., Pickles, A., Falcaro, M., Marsden, A. J., Happé, F., Scott, S. K., ... & Charman, T. (2011). A multimodal approach to emotion recognition ability in autism spectrum disorders. Journal of Child Psychology and Psychiatry,52(3), 275-285. \| *not reported \| Parametric \|  \| \| \| \| Kirchner, J. C., Hatri, A., Heekeren, H. R., & Dziobek, I. (2011). Autistic symptomatology, face processing abilities, and eye fixation patterns. Journal of autism and developmental disorders, 41(2), 158-167. \| Yes \| Parametric \| \| not reported \| \| \| *Lacroix, A., Guidetti, M., & Reilly, J. (2009). Recognition of emotional and nonemotional facial expressions: A comparison between Williams syndrome and autism. Research in developmental disabilities. \| Yes \| Parametric \| not reported \| \| \| \| *Lindner, J. L., & Rosén, L. A. (2006). Decoding of emotion through facial expression, prosody and verbal content in children and adolescents with Asperger’s syndrome. Journal of Autism and Developmental Disorders, 36(6), 769-777. \| No \| Parametric \| \| \| \| \| *Macdonald, H., Rutter, M., Howlin, P., Rios, P., Conteur, A. L., Evered, C., & Folstein, S. (1989). Recognition and expression of emotional cues by autistic and normal adults. Journal of Child Psychology and Psychiatry, 30(6), 865-877. \| Yes \|  \|  \| \| \| \| O’Connor, K. (2007). Brief report: Impaired identification of discrepancies between expressive faces and voices in adults with Asperger’s syndrome.Journal of autism and developmental disorders, 37(10), 2008-2013. \| No \| Parametric \| \| \| \| \| *Ozonoff, S., Pennington, B. F., & Rogers, S. J. (1990). Are there emotion perception deficits in young autistic children?. Journal of Child Psychology and Psychiatry, 31(3), 343-361. \| No \| Parametric \| \| \| \| \| Pelphrey, K. A., Sasson, N. J., Reznick, J. S., Paul, G., Goldman, B. D., & Piven, J. (2002). Visual scanning of faces in autism. Journal of autism and developmental disorders, 32(4), 249-261. \| No \| Parametric \| \|  \| \| \| *Philip, R. C. M., Whalley, H. C., Stanfield, A. C., Sprengelmeyer, R., Santos, I. M., Young, A. W., ... & Hall, J. (2010). Deficits in facial, body movement and vocal emotional processing in autism spectrum disorders. Psychological medicine, 40(11), 1919-1929. \| Yes \| Parametric \| \| Normal \| \| \| *Piggot, J., Kwon, H., Mobbs, D., Blasey, C., Lotspeich, L., Menon, V., ... & Reiss, A. L. (2004). Emotional attribution in high-functioning individuals with autistic spectrum disorder: a functional imaging study. Journal of the American Academy of Child & Adolescent Psychiatry, 43(4), 473-480. \| Yes \| Parametric (log-transformed) \| \| Unclear \| \| \| Robel, L., Ennouri, K., Piana, H., Vaivre-Douret, L., Perier, A., Flament, M. F., & Mouren-Siméoni, M. C. (2004). Discrimination of face identities and expressions in children with autism: Same or different?. European child & adolescent psychiatry, 13(4), 227-233. \| No \| Non-parametric \| \| \| \| \| *Rosset, D. B., Rondan, C., Da Fonseca, D., Santos, A., Assouline, B., & Deruelle, C. (2008). Typical emotion processing for cartoon but not for real faces in children with autistic spectrum disorders. Journal of autism and developmental disorders, 38(5), 919-925. \| No \| Parametric \| \|  \| \| \| Rump, K. M., Giovannelli, J. L., Minshew, N. J., & Strauss, M. S. (2009). The development of emotion recognition in individuals with autism. Child development, 80(5), 1434-1447. \| No \| Parametric \| \|  \| \| \| Rutherford, M. D., & Towns, A. M. (2008). Scan path differences and similarities during emotion perception in those with and without autism spectrum disorders. Journal of autism and developmental disorders, 38(7), 1371-1381. \| No \| Parametric \| \|  \| \| \| Sawyer, A. C., Williamson, P., & Young, R. L. (2012). Can Gaze Avoidance Explain Why Individuals with Asperger’s Syndrome Can’t Recognise Emotions From Facial Expressions?. Journal of autism and developmental disorders,42(4), 606-618. \| No \| Parametric \| \|  \| \| \| Spezio, M. L., Adolphs, R., Hurley, R. S., & Piven, J. (2007). Abnormal use of facial information in high-functioning autism. Journal of autism and developmental disorders, 37(5), 929-939. \| No \| Non-parametric \| \| \| \| \| *Tantam, D., Monaghan, L., Nicholson, H., & Stirling, J. (2006). Autistic children's ability to interpret faces: a research note. Journal of Child Psychology and Psychiatry, 30(4), 623-630. \| Yes \| Parametric \| \| No reported \| \| \| Tracy, J. L., Robins, R. W., Schriber, R. A., & Solomon, M. (2011). Is emotion recognition impaired in individuals with autism spectrum disorders?. Journal of autism and developmental disorders, 41(1), 102-109. \| No \| Parametric \| \|  \| \| \| Wallace, G. L., Case, L. K., Harms, M. B., Silvers, J. A., Kenworthy, L., & Martin, A. (2011). Diminished sensitivity to sad facial expressions in high functioning autism spectrum disorders is associated with symptomatology and adaptive functioning. Journal of autism and developmental disorders, 41(11), 1475-1486. \| No \| Non-parametric \| \| \| \| \| Wallace, S., Coleman, M., & Bailey, A. (2008). An investigation of basic facial expression recognition in autism spectrum disorders. Cognition and Emotion, 22, 1353–1380. \| Yes \| Parametric \| \| Gaussian/Normal \| \| \| Wicker, B., Fonlupt, P., Hubert, B., Tardif, C., Gepner, B., & Deruelle, C. (2008). Abnormal cerebral effective connectivity during explicit emotional processing in adults with autism spectrum disorder. Social cognitive and affective neuroscience, 3(2), 135-143. \| No \| Parametric \| \|  \| \| \| *Wright, B., Clarke, N., Jordan, J. O., Young, A. W., Clarke, P., Miles, J., ... & Williams, C. (2008). Emotion recognition in faces and the use of visual context Vo in young people with high-functioning autism spectrum disorders. Autism,12(6), 607-626. \| No \| Parametric \| \|  \| \| |
| --- | --- | --- | --- | --- | --- | --- | --- | --- | --- | --- | --- | --- | --- | --- | --- | --- | --- | --- | --- | --- | --- | --- | --- | --- | --- | --- | --- | --- | --- | --- | --- | --- | --- | --- | --- | --- | --- | --- | --- | --- | --- | --- | --- | --- | --- | --- | --- | --- | --- | --- | --- | --- | --- | --- | --- | --- | --- | --- | --- | --- | --- | --- | --- | --- | --- | --- | --- | --- | --- | --- | --- | --- | --- | --- | --- | --- | --- | --- | --- | --- | --- | --- | --- | --- | --- | --- | --- | --- | --- | --- | --- | --- | --- | --- | --- | --- | --- | --- | --- | --- | --- | --- | --- | --- | --- | --- | --- | --- | --- | --- | --- | --- | --- | --- | --- | --- | --- | --- | --- | --- | --- | --- | --- | --- | --- | --- | --- | --- | --- | --- | --- | --- | --- | --- | --- | --- | --- | --- | --- | --- | --- | --- | --- | --- | --- | --- | --- | --- | --- | --- | --- | --- | --- | --- | --- | --- | --- | --- | --- | --- | --- | --- | --- | --- | --- | --- | --- | --- | --- | --- | --- | --- | --- | --- | --- | --- | --- | --- | --- | --- | --- | --- | --- | --- | --- | --- | --- | --- | --- | --- | --- | --- | --- | --- | --- | --- | --- | --- | --- | --- | --- | --- | --- | --- | --- | --- | --- | --- | --- | --- | --- | --- | --- | --- | --- | --- | --- | --- | --- | --- | --- | --- | --- | --- | --- | --- | --- | --- | --- | --- | --- | --- | --- | --- | --- | --- | --- | --- | --- | --- | --- | --- | --- | --- | --- | --- | --- | --- | --- | --- | --- | --- | --- | --- | --- | --- | --- | --- | --- | --- | --- | --- | --- | --- | --- | --- | --- | --- | --- | --- | --- | --- | --- | --- | --- | --- | --- | --- | --- | --- | --- | --- | --- | --- | --- | --- | --- | --- |

**Reference**

1. Uljarevic M, Hamilton A. Recognition of emotions in autism: a formal meta-analysis. J Autism Dev Disord. 2013;43: 1517–1526. doi:10.1007/s10803-012-1695-5
