# Supplementary figures and images for: The meaning of significant mean group differences for biomarker discovery
Source: PLoS Comput Biol. 2021 Nov 18;17(11):e1009477. doi: 10.1371/journal.pcbi.1009477 (PMC8601419; doi:10.1371/journal.pcbi.1009477)

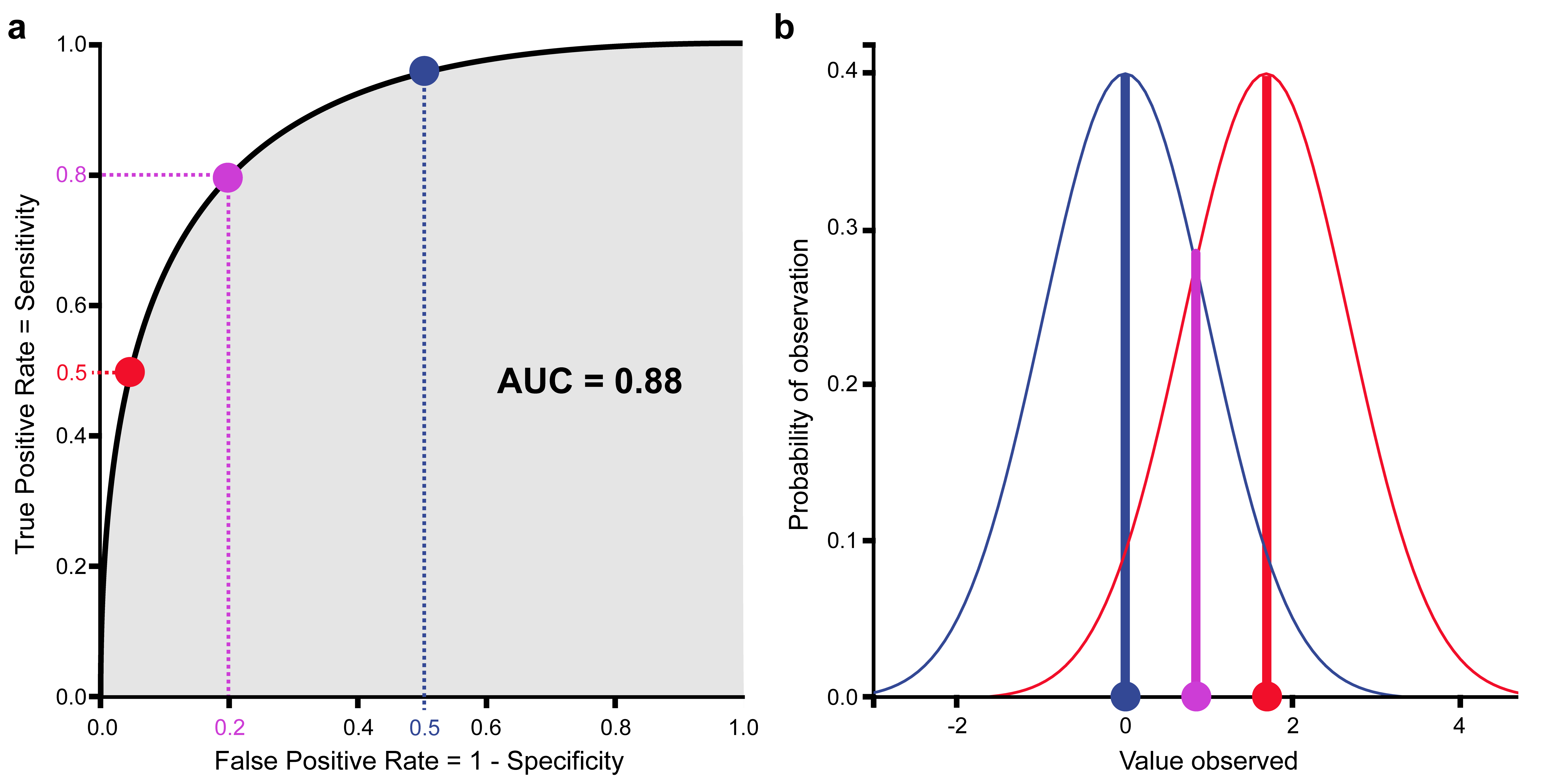

Supplement: S1 Fig — The coloured circles indicate key thresholds at half the control group distribution (blue; i.e., 0.5 specificity), at the best separation between the groups (purple; 80% sensitivity, 80% specificity), and at half of the patient distribution (red; i.e., 50% sensitivity). Inspired by the ROC curve interactive demonstration http://arogozhnikov.github.io/2015/10/05/roc-curve.html. AUC, area under the curve; ROC curve, receiver operating characteristic curve. (TIF) [file pcbi.1009477.s004.tif]
