# Supplementary material for: The meaning of significant mean group differences for biomarker discovery
Source: PLoS Comput Biol. 2021 Nov 18;17(11):e1009477. doi: 10.1371/journal.pcbi.1009477 (PMC8601419; doi:10.1371/journal.pcbi.1009477)
Supplement: S1 Text — Python (Python Software Foundation; https://www.python.org/) was selected as the scientific computing engine. Capitalising on its open-source ecosystem helps enhance replicability, reusability, and provenance tracking. The Numpy (van der Walt and colleagues, 2011), Scipy (Virtanen and colleagues, 2020), and Matplotlib (Hunter, 2007) packages were used to generate all numerical simulations. Scripts that reproduce the results of the present study are readily accessible and open for reuse (https://gist.github.com/deep-introspection/4280aeee34a0f1ab4491a386adcd5dad/). We generated 2 populations with varying sample sizes per group (20 and 100). (DOCX) [file pcbi.1009477.s006.docx]

**S1 Text. Scientific computing and empirical simulations.**

Python (Python Software Foundation, <https://www.python.org/>) was selected as the scientific computing engine. Capitalizing on its open-source ecosystem helps enhance replicability, reusability, and provenance tracking. The Numpy [1], Scipy [2], and Matplotlib [3] packages were used to generate all numerical simulations. Scripts that reproduce the results of the present study are readily accessible and open for reuse (<https://gist.github.com/deep-introspection/4280aeee34a0f1ab4491a386adcd5dad/>). We generated two populations with varying sample sizes per group (20 and 100).

**References**

1. van der Walt S, Colbert SC, Varoquaux G. The NumPy Array: A Structure for Efficient Numerical Computation. Comput Sci Eng. 2011;13: 22–30. doi:10.1109/MCSE.2011.37

2. Virtanen P, Gommers R, Oliphant TE, Haberland M, Reddy T, Cournapeau D, et al. SciPy 1.0: fundamental algorithms for scientific computing in Python. Nat Methods. 2020; 1–12. doi:10.1038/s41592-019-0686-2

3. Hunter JD. Matplotlib: A 2D Graphics Environment. Comput Sci Eng. 2007;9: 90–95. doi:10.1109/MCSE.2007.55
