# Supplementary material for: The meaning of significant mean group differences for biomarker discovery
Source: PLoS Comput Biol. 2021 Nov 18;17(11):e1009477. doi: 10.1371/journal.pcbi.1009477 (PMC8601419; doi:10.1371/journal.pcbi.1009477)
Supplement: S2 Text — Here, we review how we can calculate the frequency and severity of atypicalities when the data of one or both groups are nonnormally distributed. The degree to which 2 distributions overlap depends both on the differences between the central tendencies and the shape of the distributions. Previously, we used the mean and SD of the comparison group as reference points to estimate how far a given individual diverges from the typical range. Cohen’s d was used as an index of the magnitude of the group separation. In normal distributions, the mean and median are the same and represent the most typical value in the data set. However, in skewed or gamma distributions, the mean is dragged more into the direction of the skew (“longer tail”) than is the median. In many instances of skewed distributions, the median is therefore the more appropriate central tendency as it characterises where the majority of individuals scored. There are a number of effect size measures available that are more “robust” to skewness, such as the scaled/unscaled robust d or the common language effect size (Li and colleagues, 2016). However, our primary interest is not in an index of the magnitude of the effect per se, but in finding a way to ascertain the frequency and severity of atypicalities on a test/measure in a clinical group. This requires us to move to nonparametric statistics. Therefore, we translated the central tendencies of means and SDs into their nonparametric counterparts of median and percentiles. The IQR is calculated by dividing the data set into 4 equal portions and refers to the “middle 50%,” i.e., the range between the 25th percentile (Q1) and the 75% percentile (Q3). The 50th percentile or (Q2) is then the same as the median. The IQR is somewhat narrower than 1 SD. The equivalent to 1 SD (68% of values) of the mean would be 68% around the median. This now provides us with a universal way to express frequencies and severities across different types of distributions. IQR, interqu [file pcbi.1009477.s007.docx]

**S2 Text. Translating means and standard deviations into non-parametric equivalents to assess the frequency and severity of atypicalities in non-normal distributions**

Here we review how we can calculate the frequency and severity of atypicalities when the data of one or both groups are non-normally distributed.

The degree to which two distributions overlap depends both on the differences between the central tendencies *and* the shape of the distributions. Previously, we used the mean and standard deviation of the comparison group as reference points to estimate how far a given individual diverges from the typical range. Cohen’s *d* was used as an index of the magnitude of the group separation. In normal distributions, the mean and median are the same and represent the most typical value in the data set. However, in skewed or gamma distributions, the mean is dragged more into the direction of the skew (“longer tail”) than is the median. In many instances of skewed distributions, the median is therefore the more appropriate central tendency as it characterizes where the majority of individuals scored.

There are a number of effect size measures available that are more ‘robust’ to skewness, such as the scaled/ unscaled robust *d* or the common language effect size [1]. However, our primary interest is not in an index of the magnitude of the effect per se, but in finding a way to ascertain the frequency and severity of atypicalities on a test/ measure in a clinical group. This requires us to move to non-parametric statistics. Therefore, we translated the central tendencies of means and standard deviations into their non-parametric counterparts of median and percentiles. The interquartile range (IQR) is calculated by dividing the data set into four equal portions and refers to the ‘middle 50%’, i.e., the range between the 25^th^ percentile (Q1) and the 75% percentile (Q3). The 50^th^ percentile or (Q2) is then the same as the median. The interquartile range is somewhat narrower than 1 standard deviation. The equivalent to one standard deviation (68% of values) of the mean would be 68% around the median. This now provides us with a universal way to express frequencies and severities across different types of distributions.

**Reference**

1. Li JC-H. Effect size measures in a two-independent-samples case with nonnormal and nonhomogeneous data. Behav Res Methods. 2016;48: 1560–1574. doi:10.3758/s13428-015-0667-z
